# The Role of Virtual and Augmented Reality in Advancing Drug Discovery in Dermatology

**DOI:** 10.1111/jocd.70071

**Published:** 2025-02-19

**Authors:** Leon Kircik, Mohamad Goldust

**Affiliations:** ^1^ 5Icahn School of Medicine at Mount Sinai New York New York USA; ^2^ Indiana University School of Medicine Indianapolis Indiana USA; ^3^ Department of Dermatology Yale University School of Medicine New Haven Connecticut USA

**Keywords:** augmented reality, dermatology, drug discovery, virtual reality

Dear Editor,

Virtual and augmented reality (VR/AR) technologies are expanding various medical fields, and their application in dermatology is gaining momentum [1, 2]. These advanced tools have proven effective in enhancing patient education, surgical planning, and diagnostics, but their potential to transform dermatological drug discovery is particularly remarkable. This letter aims to provide an overview of how VR/AR can accelerate drug development, personalize treatments, and improve patient outcomes while addressing key challenges and highlighting real‐world examples.

## 1 Understanding VR and AR in Medicine

Virtual reality (VR) creates immersive digital environments that simulate real‐world scenarios, allowing users to interact with and manipulate virtual objects. Augmented reality (AR) overlays digital information onto the real world, enhancing the user's perception of their environment. These technologies are increasingly being adopted in medicine for applications such as surgical simulations and medical training. In dermatology, VR and AR can provide valuable insights into complex skin diseases and drug interactions, leading the way for innovative therapeutic strategies.

## 2 Applications in Dermatological Drug Discovery

The traditional drug discovery process is lengthy, expensive, and resource‐intensive. VR/AR technologies offer a promising alternative by enabling researchers to visualize and interact with detailed 3D models of skin anatomy, cellular structures, and molecular interactions. For instance, VR can recreate disease models, such as psoriasis or eczema, allowing researchers to simulate drug responses in a controlled virtual environment. This capability reduces reliance on animal models and traditional wet‐lab experiments, facilitating preclinical research.

One practical example is the use of AR to visualize molecular docking in drug discovery. AR applications, such as Molecular Rift, enable scientists to interact with 3D molecular models, facilitating a better understanding of drug‐target interactions. By using AR to simulate drug penetration and cellular response, researchers can optimize compound formulations before proceeding to costly clinical trials [3].

## 3 Advancing Personalized Medicine

The variability in skin structure and function among individuals highlights the need for personalized treatments in dermatology. VR/AR technologies can create patient‐specific 3D models derived from imaging data, such as biopsies. These models allow researchers to simulate drug interactions with unique skin compositions, helping to identify the most effective therapies for individual patients. For example, VR systems like Nanome provide platforms for collaborative exploration of molecular interactions, enabling personalized drug design based on patient‐specific data [4].

## 4 Improving Clinical Trials

VR/AR technologies also hold promise for optimizing clinical trial design and execution. AR can monitor treatment effects by overlaying digital data onto patients' skin, enabling real‐time visualization of lesion changes and drug penetration. Tools like Microsoft's HoloLens have been employed in medical research to visualize and track disease progression. Additionally, VR can enhance patient engagement by offering educational modules that improve the understanding of trial procedures, thereby increasing recruitment and retention rates.

## 5 Regulatory and Ethical Considerations

The integration of VR/AR into dermatological practice raises several regulatory and ethical challenges. Data privacy and informed consent are critical concerns, especially when using patient‐specific models. Ensuring equitable access to these technologies is essential to prevent disparities in healthcare delivery. Regulatory frameworks must evolve to address these issues, providing guidelines for the safe and effective implementation of VR/AR in clinical practice [5].

## 6 Real‐World Applications and Future Directions

While the use of VR/AR in dermatological drug discovery is still in its early stages, practical applications are emerging. For example, VR platforms are being used to train dermatologists in complex procedures, while AR tools assist in visualizing treatment outcomes. Companies like VRHealth and EchoPixel are actively developing VR/AR solutions for medical applications, demonstrating the feasibility of these technologies in real‐world settings.

Collaboration between dermatologists, researchers, and technologists will be crucial to utilize the full potential of VR/AR in drug discovery. By addressing technical challenges, such as creating high‐fidelity virtual models of skin diseases and overcoming regulatory challenges, these technologies can become integral to dermatological research and treatment.

In conclusion, VR and AR represent transformative tools for dermatological drug discovery. By enhancing preclinical research, personalizing therapies, and optimizing clinical trials, these technologies have the potential to reform treatment for skin diseases. Continued research and collaboration will ensure their successful integration into dermatology, leading the way for innovative solutions and improved patient outcomes.

## Disclosure

We confirm that the manuscript has been read and approved by all the authors, that the requirements for authorship as stated earlier in this document have been met and that each author believes that the manuscript represents honest work.

## Consent

The authors have nothing to report.

## Conflicts of Interest

The authors declare no conflicts of interest.

## Data Availability

Data sharing not applicable to this article as no datasets were generated or analysed during the current study.
